# Titania/chitosan–lignin nanocomposite as an efficient photocatalyst for the selective oxidation of benzyl alcohol under UV and visible light†

**DOI:** 10.1039/d1ra06500a

**Published:** 2021-10-28

**Authors:** Ayesha Khan, Michael Goepel, Wojciech Lisowski, Dariusz Łomot, Dmytro Lisovytskiy, Marta Mazurkiewicz-Pawlicka, Roger Gläser, Juan Carlos Colmenares

**Affiliations:** a Institute of Physical Chemistry, Polish Academy of Sciences Warsaw 01-224 Poland akhan@ichf.edu.pl jcarloscolmenares@ichf.edu.pl; b Institute of Chemical Technology, Leipzig University Leipzig 04103 Germany roger.glaeser@uni-leipzig.de; c Faculty of Chemical and Process Engineering, Warsaw University of Technology Warsaw 00-645 Poland

## Abstract

Developing functional materials from biomass is a significant research subject due to its unique structure, abundant availability, biodegradability and low cost. A series of chitosan–lignin (CL) composites were prepared through a hydrothermal method by varying the weight ratio of chitosan and lignin. Subsequently, these CL composites were combined with titania (T) to form a nanocomposite (T/CL) using sol–gel and hydrothermal based methods. T/CL nanocomposites exhibited improved photocatalytic performance in comparison with sol–gel and hydrothermally prepared pristine titania (SGH-TiO_2_), towards the selective oxidation of benzyl alcohol (BnOH) to benzaldehyde (Bnald) under UV (375 nm) and visible light (515 nm). More specifically, the 75T/CL(25 : 75) nanocomposite (a representative photocatalyst from the 75T/CL nanocomposite series) showed very high selectivity (94%) towards Bnald at 55% BnOH conversion under UV light. Whereas, SGH-TiO_2_ titania exhibited much lower (68%) selectivity for Bnald at similar BnOH conversion. Moreover, the 75T/CL(25 : 75) nanocomposite also showed excellent Bnald selectivity (100%) at moderate BnOH conversion (19%) under visible light. Whereas, SGH-TiO_2_ did not show any activity for BnOH oxidation under visible light. XPS studies suggest that the visible light activity of the 75T/CL(25 : 75) nanocomposite is possibly related to the doping of nitrogen into titania from chitosan. However, according to UV-visible-DRS results, no direct evidence pertaining to the decrease in band-gap energy of titania was found upon coupling with the CL composite and the visible light activity was attributed to N-doping of titania. Overall, it was found that T/CL nanocomposites enhanced the photocatalytic performance of titania *via* improved light harvesting and higher selectivity through mediation of active radical species.

## Abstract 2

Combining titania with chitosan–lignin composites results in an active and selective photocatalyst for the oxidation of benzyl alcohol to benzaldehyde under green light (515 nm).

## Graphical abstract

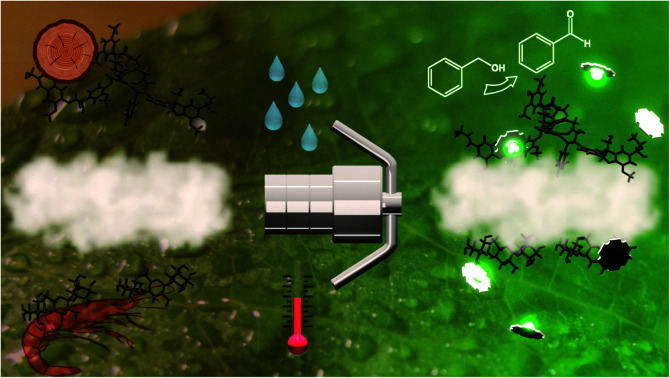

## 1. Introduction

The dwindling supply of fossil-fuel reserves and detrimental effects of fossil-fuel utilization on the environment have triggered immense research efforts in exploring renewable resources for the production of chemicals and fuels and the synthesis of functional materials.^1^ Biomass, as an inexhaustible and abundantly available carbon source, has great potential for the preparation of carbonaceous materials.^2^ The development of high-performance photocatalytic materials from biomass-derived polymers (cellulose, lignin, and chitin) is a subject of research, owing to the unique structure, functional groups, low cost, and biodegradability of these biopolymers.^3^ Additionally, it is an attractive approach to reduce the carbon-footprint of biomass transformation processes. Interestingly, biomass-derived carbonaceous materials can play a dual role as catalyst and catalyst carrier in photocatalytic reactions due to their high electron conductivity, chemical inertness, and suitable surface and textural properties.^4^ Moreover, carbonaceous materials can be chemically functionalized or coupled with metal/metal oxide catalysts to impart or enhance the catalytic activity.^4^ In particular, their application in the form of supports or composites with active metal/metal oxide catalysts has attracted attention in liquid and gas-phase reactions.^5^

Among the numerous sources of carbonaceous materials, chitosan (derived from the partial deacetylation of chitin) is a nitrogen-rich (∼7 wt%) copolymer, made up of repeating units of *N*-acetyl-d-glucosamine and d-glucosamine.^1^ There has been growing interest in the use of chitosan for the preparation of, supported photocatalysts, and composites, attributed to the presence of amine and hydroxyl functional groups which may interact with various metal oxides such as titania,^6^ zinc oxide,^7^ zeolite,^8^*etc.* Saravanan *et al.*^9^ prepared titania–chitosan (TiO_2_/CS) nanocomposites for the photocatalytic degradation of methyl orange (MO). The TiO_2_/CS nanocomposite prepared in the weight ratio of 75 : 25 showed 63.5% degradation of MO after 120 minutes, under simulated solar irradiation. Whereas, pristine titania was found to be inactive for the degradation of MO. The visible light activity of TiO_2_/CS nanocomposite is ascribed to its slightly reduced (3.0 eV) band gap compared to pristine titania (3.2 eV).^9^ Besides that, chitosan may facilitate the nitrogen doping and can possibly improve the visible light harvesting of a photocatalyst.^1^ Lignin, another macromolecular polymer (derived from lignocellulosic waste) with multiple functional groups (hydroxyl, methoxy, ether, and aldehyde groups)^10^ is another potential candidate for the preparation of composite materials.^11^ Besides chemical modification, there are a couple of other advantages associated with lignin for its application in the development of photocatalytic materials. For example, the phenolic groups of the lignin possibly improve the hole transport features of the materials.^12,13^ Whereas, the aromatic structure and the chromophore groups present in lignin allow it to absorb solar radiation,^14,15^ especially in the range of 295–400 nm.^16^ Moreover, lignin may undergo a photoinduced electron transfer involving molecular oxygen and other substrate species, which result in the formation of reactive oxygen species (ROS) such as the superoxide radical anion (O_2_˙^−^) and hydroxyl radical (˙OH). This enables its use as a photosensitizer for photocatalytic application.^17^

Although both biopolymers, chitosan and lignin have a number of advantageous properties, there are also some key concerns associated with the individual materials. In terms of the physical properties of chitosan, it has low mechanical and thermal stability.^18^ Additionally, it is generally insoluble in neutral and basic pH range.^19^ Whereas, the solubility of lignin in different solvents varies with the type of lignin. From this perspective, blending chitosan with another biopolymer like lignin presents the possibility to obtain a biopolymer-based composite with improved physicochemical properties. The cationic nature of chitosan in acidic medium favors the interaction with negatively charged polymer or materials. Integrating sulfonated lignin, as a counter ion polymer into chitosan may result in the formation of ionic linkages between the two components^20^ and possibly enhance the stability of the material.^21^ Therefore, preparing a composite of chitosan and lignin is a fascinating approach to overcome the limitations of individual polymer.

Chitosan–lignin (CL) composites are widely studied as adsorbents for the removal of dyes in aqueous solution.^22^ In wastewater remediation, the improved performance of CL composites is attributed to the presence of various functional moieties, which facilitate the dye adsorption.^23^ Despite the high level of interest in the application of CL composite in environmental remediation,^22,24^ their application in photocatalysis is, to the best of our knowledge, not yet documented. Interestingly, CL composites are adaptable to couple with metal or metal oxide nanoparticles owing to the presence of multiple functional groups,^23^ which make them a suitable candidate for photocatalytic application. The diverse functional moieties of CL composite, specifically aromatic groups of lignin, may enhance the adsorption of substrate due to π–π stacking interaction with organic adsorbates, which is beneficial for enhancing the activity of the photocatalyst.^25,26^ Masilompane *et al.*^23^ reported that chitosan–lignin–titania nanoadsorbent has the capacity to remove Brilliant Black (BB) dye from contaminated wastewater due to strong electrostatic attraction between BB and chitosan–lignin–titania nanocomposite. The monolayer adsorption capacities calculated was 15.8 mg g^−1^ at 25 °C using the linear Langmuir isotherm.^23^

Recently, extensive efforts are being made to carry out photocatalytic reactions using solar energy. However, to utilize solar light the development of ecofriendly visible light (accounts ∼45% of solar radiation) active photocatalysts is a challenging task. Nanostructured-titania is one of the most widely studied photocatalysts, due to its low cost, high photocatalytic activity and stability.^27^ Despite of several advantages, there are few drawbacks of titania which limits its application in certain fields of photocatalysis, especially in organic synthesis. For example, titania exhibits high recombination rate of electron–hole pairs and lacks an appropriate band-gap^28^ required for the visible light absorption (*i.e.* <3.0 eV). These intrinsic features of titania limits its application in visible-light driven photocatalysis.^29^ However, efforts have been made to improve the visible light activity of titania for the selective oxidation reactions. Higashimoto *et al.*^30,31^ reported the photocatalytic partial oxidation of benzyl alcohol (BnOH) to benzaldehyde (Bnald) under visible light (*λ* > 420 nm) *via* surface complex formation by the adsorption of BnOH on titania surface. A high conversion for BnOH (>99%) was achieved with high Bnald selectivity (>99%) in acetonitrile after 4 hours of irradiation.^30^ Li *et al.*^32^ observed similar results for the photocatalytic activity of single crystalline rutile titania nanorods for the selective oxidation of BnOH to Bnald (>99% selectivity) *via* surface complex formation under visible light (*λ* ≥ 420 nm).^32^ Moreover, constructing a heterojunction of TiO_2_ and In_2_O_3,_ and further decorating it with Pt nanoparticles and MnO_*x*_ to prepare a mesoporous hollow spheres (Pt@TiO_2_@In_2_O_3_@MnO_*x*_, PTIM-MSs) is reported to be an efficient approach for the photocatalytic oxidation of BnOH to Bnald. The PTIM-MSs exhibited high activity for the selective oxidation of BnOH, with ∼1000 μmol g^−1^ Bnald formation compared to TiO_2_ mesoporous hollow spheres (∼300 μmol g^−1^) after 14 hours of irradiation under simulated sunlight.^33^ In another study, a plasmonic photocatalyst based on Pt nanoparticles supported on anatase titania achieved high Bnald yield (72%) at 75% BnOH conversion under natural sunlight.^34^

Besides the visible light activation of titania, separation of nano-sized titania from the liquid reaction medium is difficult because of their fine size.^35,36^ Herein, we focus on preparing nanocomposites utilizing biomass-derived waste (chitosan and lignin) and titania, with the aim to improve the visible light (515 nm) activity as well as photocatalytic efficiency of titania under UV (375 nm) light for the selective oxidation of benzyl alcohol (BnOH) to benzaldehyde (Bnald). Moreover, this strategy may overcome the problem of titania nanoparticles separation and recovery from the liquid reaction medium.

## 2. Experimental

### 2.1 Materials

Medium molecular weight chitosan (Sigma-Aldrich), alkali lignin, low sulfonate content (Sigma-Aldrich), citric acid monohydrate (≥99.0%, STANLAB), titanium(iv) isopropoxide (97+%, Sigma-Aldrich), 2-propanol (99.7%, Alfa Aesar), nitric acid (65%, Alfa Aesar), acetonitrile (99.9%, POCH), methanol (99.9%, POCH), benzyl alcohol (99.8%, Sigma-Aldrich), 1,4-benzoquinone (98%, Sigma-Aldrich), dimethylsulfoxide (>99.5%, ROTH), sodium acetate (99.0% Chempur), sodium sulfate (>99%, ROTH), orthophosphoric acid (85%, POCH), potassium trioxalatoferrate(iii) trihydrate (98%, abcr), Nafion (∼5% in a mixture of lower aliphatic alcohols and water, Sigma-Aldrich), 1,10-phenanthroline (99.5%, Chempur) and active carbon Norit® SX 2 (POCH) were used without further purification. Water used was purified to 18 MΩ cm resistivity by Milli-Q water purification system.

### 2.2 Preparation of chitosan–lignin (CL) composite

CL composites were prepared in different weight ratios (10 : 90, 25 : 75, 50 : 50, 75 : 25 and 90 : 10) as follows. First, a 0.5 wt% chitosan solution was prepared in 0.2 M aqueous citric acid solution (100 mL) and stirred well at 800 rpm for 2 hours to form a homogeneous solution. A known mass of lignin was added to chitosan citric acid solution and stirred at 800 rpm for 2 hours. Finally, the brown suspension obtained was sealed into a Teflon equipped stainless steel autoclave, which was then placed in a hot air oven followed by hydrothermal treatment at 200 °C for 12 hours. After hydrothermal treatment, the autoclave was cooled down naturally. The obtained blackish brown suspension was filtered and the precipitates were washed with water and 2-propanol twice and then dried at 80 °C for 12 hours.

### 2.3 Preparation of titania sol

A titania sol was prepared by acid-catalyzed hydrolysis of titanium(iv) isopropoxide.^37^ In a typical procedure, a specified volume (9.077 mL) of titanium(iv) isopropoxide was dissolved in 2-propanol (25 mL) and stirred (400 rpm) for two hours at room temperature. Subsequently, 1 M HNO_3_ (1 mL) was added to the solution under continuous stirring for 5 minutes until gelation takes place. Then, 25 mL of water were slowly added to the gel and stirred for another three hours.

### 2.4 Synthesis of titania/chitosan–lignin (T/CL) nanocomposites

To obtain the T/CL nanocomposites with the weight fraction of 75/25 (75 wt% titania and 25 wt% CL composite), the titania sol was added to a suspension of CL composite (0.8 g) in 160 mL of deionized water at room temperature under stirring. The resulting suspension was stirred for 5 hours, then filtered, washed with water and dried at 80 °C for 12 hours. Afterwards, the obtained solid was ground and then transferred to Teflon lined autoclave filled (∼80%) with water for hydrothermal treatment at 150 °C for 8 hours. Finally, the obtained nanocomposite named 75T/CL was dried at 110 °C in an oven for 12 hours. In this manner, a series of nanocomposites were synthesized using CL composites prepared in different weight ratios (10 : 90, 25 : 75, 50 : 50, 75 : 25 and 90 : 10). For comparison, pristine titania (SGH-TiO_2_) and a nanocomposite of Norit (75T/Norit) were also prepared using the same sol–gel and hydrothermal route (in case of SGH-TiO_2,_ omitting the composite formation). Moreover, to evaluate the effect of titania content, a series of nanocomposites was also prepared by varying the titania content (50 wt%, 85 wt%, 95 wt% and 99 wt%) for the selected CL composite (CL(25 : 75)). A specified volume of titanium(iv) isopropoxide, corresponding to the definite wt% of titania (50 wt%, 85 wt%, 95 wt% and 99 wt%) was used to prepare the titania sol, and the prepared nanocomposites were labelled as 50T/CL(25 : 75), 85T/CL(25 : 75), 95T/CL(25 : 75) and 99T/CL(25 : 75), respectively.

### 2.5 Photocatalyst characterization

The morphology of the CL composites was examined by FEI Nova Nanolab 200 scanning electron microscopy (SEM) at an accelerating voltage of 15 kV. X-ray diffraction (XRD) measurements were performed employing Bragg–Brentano configuration. This type of arrangement was provided using Empyrean diffraction platform from Malvern PANalytical Co., powered at 40 kV × 40 mA and equipped with a vertical goniometer, with theta–theta geometry using Ni filtered Cu Kα radiation. Data were collected in range of 2*θ* = 9°–100°, with step size of 0.008° and counting time up to 60 second per step. The percentage phase composition was determined through the Rietveld refinements of the XRD patterns. Whereas, the average crystallite size was determined according to the Scherrer equation (eqn (1)), where *D* is the average crystallite size of the catalyst (nm), *λ* is the wavelength of the Cu kα X-ray radiation (*λ* = 0.154056 nm), *k* is a coefficient (shape factor) taken as 0.94, *β* is the full width at half maximum (FWHM) intensity of the peak observed at 2*θ* (radian), and *θ* is the diffraction angle.

1 *D* = *kλ*/*β* cos *θ*

The specific surface area and pore width distribution of the samples were determined through N_2_ physisorption isotherms by applying Brunauer–Emmet–Teller (BET) and Barrett, Joyner, Halenda (BJH) method, respectively. The measurements were carried out at Micrometrics ASAP 2020 automated system. The FTIR spectrum was recorded on Bruker ATR spectrometer in the range of 4000–400 cm^−1^ in transmittance mode with 16 scans and a resolution of 4 cm^−1^. Diffuse reflectance UV-visible spectroscopy measurements were performed using a UV/vis/NIR spectrophotometer Jasco V-570 equipped with an integrating sphere. The baseline was recorded using Spectralon™ (poly(tetrafluoroethylene)) as a reference material. Band-gaps values were calculated using Tauc plot applying Kubelka–Munk function. Elemental analysis of the samples was performed on Thermo Scientific Flash 2000 Organic Elemental Analyzer. X-ray photoelectron spectroscopy (XPS) experiments were performed in a PHl 5000 VersaProbe™ – spectrometer (ULVAC-PHI, Chigasaki Japan). The XPS spectra were recorded using monochromatic Al-Kα radiation (*hν* = 1486.6 eV) from an X-ray source operating at 100 μm spot size, 25 W and 15 kV. Both survey and high-resolution (HR) XPS spectra were collected with the analyser pass energy of 117.4 eV and 23.5 eV and the energy step size of 0.4 and 0.1 eV, respectively. Casa XPS software (v.2.3.19, Casa Software Ltd, Wilmslow, United Kingdom) was used to evaluate the XPS data. Shirley background subtraction and peak fitting with Gaussian–Lorentzian-shaped profiles was performed. The binding energy (BE) scale was referenced to the C 1s peak with BE = 284.8 ± 0.2 eV and Ti 2p_3/2_ peak with BE = 458.6 ± 0.2 eV. For quantification the PHI Multipak sensitivity factors and determined transmission function of the spectrometer were used. Thermal stability of CL composites was studied using thermogravimetric analysis (TGA) performed on Netzsch STA 409 TG/DTA device. The samples were analyzed under N_2_ atmosphere (75 mL min^−1^) with the heating ramp of 10 °C min^−1^. Whereas, TGA measurements for nanocomposites were performed under air flow (30 mL min^−1^) with the heating ramp of 20 °C min^−1^ using Mettler Toledo TGA/DSC 3+ system, to estimate the titania content. The high resolution TEM (HR-TEM) measurements were carried out on FEI Talos F200X transmission microscope at 200 kV. In order to estimate the particle size more than 200 particles were counted.

### 2.6 Photoelectrochemical measurements

The photoelectrochemical measurements were performed on an electrochemical workstation (Ivium Bipotentiostat) in a typical three-electrode system using Ag/AgCl electrode as a reference electrode and Pt wire as a counter electrode. 0.2 M sodium sulphate was employed as an electrolyte solution. The ITO electrode covered with photocatalyst served as a working electrode. The working electrode was prepared by the following method:^38^ 10 mg of the photocatalyst were mixed with 300 μL of 2-propanol, 100 μL of Milli-Q water and 20 μL of Nafion solution then treated ultrasonically for 30 minutes to obtain a suspension. Finally, the suspension (20 μL) was drop-cast onto the ITO glass surface (0.77 cm^2^) and dried in air to form the working electrode. The electrochemical impedance spectra (EIS) were recorded over the frequency range of 0.01 to 1000 Hz with a 10 mV amplitude of the AC signal at 0 V *vs.* OCP (open circuit potential). The transient photocurrent responses were measured using chronoamperometry method at 0 V *vs.* OCP using green (515 nm) LED lamp as a light source.

### 2.7 Photocatalytic selective oxidation of benzyl alcohol (BnOH)

The photocatalytic reaction was performed in a glass photoreactor (20 mL). 20 mL of 0.5 mM (0.01 mmol) BnOH solution prepared in acetonitrile and 20 mg of photocatalyst (1 g L^−1^) were charged into the photoreactor. Afterwards, the suspension was magnetically stirred (400 rpm) for 1 hour in the dark to achieve the equilibrium. The photocatalytic reactions were performed under the irradiation of UV (*λ* = 375) LED lamps and green LED lamps (*λ* = 515 nm). Each light source consists of six LEDs and the photo-intensity of the green and UV LEDs was determined to be 6 × ∼9 W m^−2^, measured by a Delta OHM HD 2302.0 light meter with a LP 471 RAD probe having a spectral range of 400–1050 nm and LP 471 UVA probe having a spectral range of 315–400 nm, respectively. The distance between the light source and the photoreactor wall was about 2 mm. At given illumination time intervals, 0.15 mL aliquots were collected, and then filtered through a nylon filter (pore size 0.2 μm) to remove the photocatalyst.

After each catalytic run the photocatalyst was collected by decanting the solvent, washed multiple times with water, dried at 110 °C for 48 hours and reused for next run with a fresh BnOH solution. Multiple catalytic runs were performed following the same procedure.

The quantitative analysis of substrate and reaction products was performed on a high-performance liquid chromatography (HPLC) instrument (Waters 2487) equipped with Sunfire C18 (4.6 × 150 mm) column using a mobile phase consisting of 77.4% Milli-Q, 20% acetonitrile, 2.5% methanol and 0.1% 0.05 M orthophosphoric acid at a flow rate of 1 mL min^−1^. The temperature of the column oven was kept at 28 °C. The BnOH conversion (eqn (2)), and Bnald selectivity (eqn (3)) were calculated as follows:2
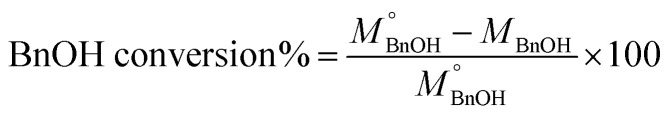
3
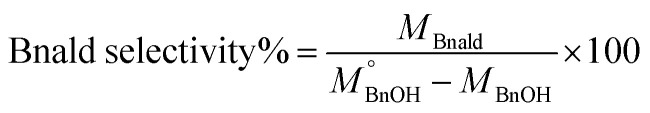
where 
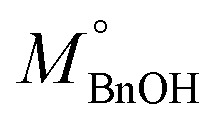
 refers to the initial amount of BnOH (mmol), whereas *M*_BnOH_ and *M*_Bnald_ corresponds to the mmol of BnOH and mmol of Bnald after the photocatalytic reaction.

### 2.8 Apparent quantum yield (AQY) measurement

The apparent quantum yield (*Φ*) for the Bnald formation is defined as the ratio of the amount of Bnald formed per unit time to the number of photons absorbed by the system per unit time (eqn (4))4
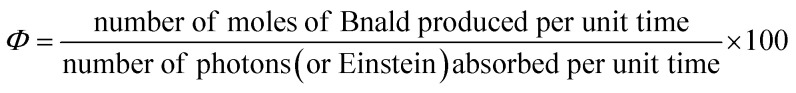


To measure the apparent quantum yield (*Φ*), the photon flux to the photoreactor was determined by potassium ferrioxalate actinometry.^39^ The experiment was performed using 0.006 and 0.15 M potassium ferrioxalate solution under UV and visible light, respectively.

### 2.9 Stability of titania/chitosan–lignin (T/CL) nanocomposite

The leaching of titanium from 75T/CL(25 : 75) nanocomposite (a representative photocatalyst) into BnOH solution after photocatalytic reaction (after 4 hours of illumination) was determined using energy dispersive X-ray fluorescence analysis (EDXRF). The EDXRF analysis was carried out using a MiniPal 4 equipment from PANalytical Co, with a Rh-tube and silicon drift detector (resolution 145 eV) to gain information on the elemental composition. The spectrum was collected in atmosphere, without using a filter, at a tube voltage of 30 kV in order to evaluate the presence of Ti. The time of acquisition was set to 600 s and the tube current up to 50 μA.

Moreover the stability of 75T/CL(25 : 75) nanocomposite (a representative photocatalyst) with respect to the degradation of chitosan and lignin was tested under dark conditions and light (UV and visible) irradiation. For this purpose, 20 mg of the photocatalyst and 20 mL of acetonitrile were charged in to a photoreactor and the resulting suspension was stirred (400 rpm) for 4 hours under dark conditions. Same experiments were performed under UV and visible light to assess the photostability of the 75T/CL(25 : 75) nanocomposite. After the experiments, 2 mL aliquots of reaction solution were collected, and then filtered through a nylon filter (pore size 0.2 μm) to remove the photocatalyst. Finally, UV-visible absorption spectra were recorded (Thermo Scientific Evolution 220, UV-vis Spectrophotometer) in the range of 200–800 nm, for the filtrate obtained.

## 3. Results and discussion

### 3.1 Characterization of chitosan–lignin (CL) composite

The as-prepared chitosan–lignin (CL) composite were characterized using various techniques and the characteristic features of the CL composites were compared with commercial activated carbon (Norit). Scanning electron microscopy (SEM) images revealed that the CL composites lack ordered surface structure and exhibited rough and non-uniform surface. Whereas, Norit exhibited irregular porous surface structure (Fig. S1, ESI†). Elemental analysis was carried out to gain information about the chemical composition of chitosan, lignin, CL composites and Norit (Table 1). Chitosan (Table 1, entry 1) contains high nitrogen content (6.2 wt%) owing to the presence of nitrogen-containing functional groups (amine and acetamide). Whereas, nitrogen was not detected in lignin and Norit. The nitrogen content in the CL composites increased with the increase in chitosan proportion in the composite (Table 1, entry 3–7). Moreover, lignin contains ∼4 wt% sulphur due to the presence of sulfonate group. The sulphur content decreased with the decrease in lignin proportion in the CL composites (Table 1, entry 3–7). Moreover, traces of sulphur were also observed in Norit (Table 1, entry 8).

**Table 1:** Elemental (CHNS wt%) composition of chitosan, lignin, CL composites and Norit

Entries	Samples	N/%	C/%	H/%	S/%
1	Chitosan	6.26	40.71	6.64	aND
2	Lignin	aND	48.26	4.73	4.16
3	CL(10 : 90)	0.06	62.47	4.98	2.06
4	CL(25 : 75)	0.29	60.59	4.83	1.62
5	CL(50 : 50)	0.55	62.84	4.75	1.25
6	CL(75 : 25)	0.76	64.77	4.7	0.89
7	CL(90 : 10)	1.28	64.93	4.65	0.44
8	Norit	aND	84.41	0.43	0.03
9	75T/CL(10 : 90)	aND	15.45	1.67	0.52
10	75T/CL(25 : 75)	aND	15.00	1.59	0.35
11	75T/CL(50 : 50)	aND	13.79	1.44	0.25
12	75T/CL(75 : 25)	aND	15.82	1.52	0.24
13	75T/CL(90 : 10)	aND	15.87	1.44	0.07
14	75T/Norit	aND	20.18	0.56	aND

a ND: not detected.

N_2_ adsorption–desorption isotherms recorded for CL composites and commercial activated carbon (Norit) can be classified to Type II isotherms and Type IV isotherm, respectively (Fig. S2, ESI†). The CL composites lack porosity and exhibited a specific surface area in the range of 10–16 m^2^ g^−1^ (Table 2, entries 1–5). Whereas, Norit was found to be mesoporous in nature with comparatively high specific surface area (558 m^2^ g^−1^). Thermogravimetric analysis (TGA) has been carried out to study the thermal degradation of CL composites and individual biopolymers under nitrogen atmosphere. Interestingly, significant changes in the shape of TGA curves of CL composites have been observed, compared to chitosan and lignin (Fig. S3, ESI†). TGA curves of the CL composites showed increased thermal stability within the range of 100–500 °C compared to chitosan and lignin, that is indicative of the interaction of the components of composite. Whereas, Norit is thermally much more stable than chitosan, lignin and CL composites (Fig. S3, ESI†). FTIR analysis was carried out to explore the functional groups of CL composites and Norit. For Norit, no noticeable IR bands were observed except at 2113 cm^−1^, which corresponds for adsorbed carbon monoxide. Whereas, the FTIR spectrum of CL composites showed some distinctive features (Fig. S4, ESI†), which is indicative of the interaction of chitosan and lignin. The band corresponds for N–H stretching vibrations (3300–3400 cm^−1^) and O–H stretching vibrations (3200–3550) in parent materials were completely disappeared in CL composites, which suggests the possible interaction of the chitosan and lignin *via* a hydroxyl group. Additionally, the band appeared around 1702 cm^−1^ for C=O stretching vibrations may ascribe to the shift in amide I band present in chitosan at 1648 cm^−1^ or presence of citric acid in the CL composite. Besides that, the bands correspond to aromatic ring vibrations at 1605, 1506, 1456 cm^−1^ in lignin were retained in CL composites. However, their intensity decreased with the decrease in lignin content in the composite. Whereas, the band for aryl ether linkages at 1260 cm^−1^, in lignin disappeared in CL composite, which indicates the cleavage of ether linkages during composite formation. Furthermore, the band related to alkyl substituted ether linkages in lignin at 1036 cm^−1^ has substantially decreased in intensity with the decrease in the amount of lignin in the composite.

**Table 2:** Textural (Brunauer–Emmett–Teller specific surface area (*S*_BET_), Barrett–Joyner–Halenda pore volume (BJH *V*_p_) and pore width (BJH *w*_p_)) and crystallographic properties of the samples

Entries	Samples	*S*_BET_/[m^2^ g^−1^]	BJH *V*_p_/[cm^3^ g^−1^]	BJH *w*_p_/[nm]	Ratio of crystalline phases	Crystal size	
					Anatase : brookite/%	Anatase/nm	Brookite/nm
1	CL(10 : 90)	10	NA	NA	NA	NA	NA
2	CL(25 : 75)	16	NA	NA	NA	NA	NA
3	CL(50 : 50)	11	NA	NA	NA	NA	NA
4	CL(75 : 25)	12	NA	NA	NA	NA	NA
5	CL(90 : 10)	9	NA	NA	NA	NA	NA
6	aNorit	558	0.40	5	NA	NA	NA
7	SGH-TiO_2_	177	0.20	3	74 : 26	5	6
8	75T/CL(10 : 90)	162	0.32	6	83 : 17	5	6
9	75T/CL(25 : 75)	174	0.23	3	79 : 21	5	6
10	75T/CL(50 : 50)	170	0.23	4	78 : 22	5	6
11	75T/CL(75 : 25)	169	0.19	3	81 : 19	5	6
12	75T/CL(90 : 10)	164	0.18	3	74 : 26	5	5
13	a75T/Norit	239	0.35	5	66 : 30	6	8

a Sample contain traces of silica.

To elucidate further the distinct surface functionality of CL composites and Norit, XPS analysis was carried out (Fig. S5a–d, ESI†). Taking CL(25 : 75) composite as a typical example, the deconvolution of N 1s spectrum of composite resolved to two components (Fig. S5c, ESI†), which corresponds for amine (399.1 eV) and amide (400.8 eV) groups, ascribed to the presence of chitosan. Whereas, nitrogen containing surface functional groups were not detected in Norit. Besides that, some sulphur containing functional groups were also detected in the CL(25 : 75) composite, as low sulfonate alkali lignin has been used for the synthesis of composite. The peaks observed (Fig. S5d, ESI†) at 162.7 eV and 163.9 eV are related to the S 2p_3/2_ and S 2p_1/2_ of the S^2−^ group, respectively. Whereas, the peaks at 166.9 eV and 168.2 eV assigned to an alkyl sulfonate group (Fig. S5d, ESI†). While, in Norit sulphur containing surface functional groups were not observed, though the elemental analysis showed the traces of sulphur in Norit. XPS analysis revealed the distinct differences in the surface functional groups of Norit and CL composite, which can play an important role in determining the properties of the nanocomposite prepared using titania nanoparticles and CL composites or Norit.

### 3.2 Characterization of titania/chitosan–lignin (T/CL) nanocomposite

**Fig. 1:**
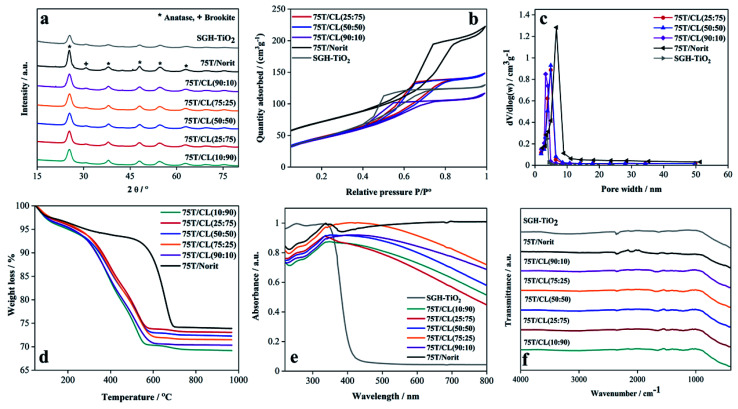
(a) X-ray diffraction (XRD) patterns of SGH-TiO_2_, 75T/CL nanocomposites and 75T/Norit nanocomposite. (b) Nitrogen adsorption–desorption isotherms of SGH-TiO_2_, 75T/CL nanocomposites and 75T/Norit nanocomposite. (c) Pore width distribution in SGH-TiO_2_, 75T/CL nanocomposites and 75T/Norit nanocomposite. (d) Thermogravimetric analysis (TGA) curves of 75T/CL nanocomposites and 75T/Norit nanocomposite. (e) UV-visible-DRS absorption spectra of SGH-TiO_2_, 75T/CL nanocomposites and 75T/Norit nanocomposite. (f) FTIR spectra of SGH-TiO_2_, 75T/CL nanocomposites and 75T/Norit nanocomposite.

The activated carbon (Norit) and as-prepared CL composites were then used to develop a series of nanocomposites based on titania nanoparticles. The characteristic features of the CL-based nanocomposites (75T/CL) were compared with activated carbon (Norit)-based nanocomposites (75T/Norit). Activated carbon is widely used as one of the benchmark material to support titania nanoparticles or for the preparation of composite with titania for the photocatalytic applications because of its high porosity, good adsorption, photosensitizing effect and low cost.^40^ The phase composition and crystal size of pristine titania, 75T/CL nanocomposites and 75T/Norit nanocomposites was investigated *via* X-ray diffraction (XRD) analysis. As shown in Fig. 1a, pristine titania, 75T/CL nanocomposites and 75T/Norit nanocomposite exhibited similar X-ray diffraction pattern. The XRD reflexes observed at 25.4° (101), 37.9° (004), 48.0° (200), 54.4° (105), 63.2° (204), indexed to the anatase phase of titania (JCPDS card no. 21-1272).^41^ Whereas, the XRD reflex observed at 30.8° (121) corresponds to the brookite phase of titania (JCPDS no. 29-1360).^42^ Moreover, the crystal size and phase composition for the pristine titania, 75T/CL nanocomposites and 75T/Norit nanocomposites is summarized in Table 2.

N_2_ adsorption–desorption isotherms were recorded to analyze the specific surface area and porosity of the nanocomposites. As presented in Fig. 1b, 75T/CL nanocomposites, 75T/Norit nanocomposites and pristine titania exhibited Type IV isotherm with H3 hysteresis which is a characteristic of mesoporous materials. The BET specific surface area observed for 75T/CL nanocomposites was in the range of 162–174 m^2^ g^−1^ (Table 2, entries 8–12), that is comparable to the specific surface area of SGH-TiO_2_ (177 m^2^ g^−1^). Whereas, 75T/Norit nanocomposite showed little higher specific surface area (269 m^2^ g^−1^) compared to 75T/CL nanocomposites and SGH-TiO_2_, which may ascribe to the higher specific surface area of Norit (Table 2, entry 6). Pore width distribution analysis (Fig. 1c) revealed that 75T/CL nanocomposites, 75T/Norit nanocomposite and SGH-TiO_2_ are mesoporous in nature (Table 2, and entry 7–13). The actual titania content in the nanocomposites was estimated by TGA measurements. As shown in Fig. 1d, the % weight loss observed for nanocomposites during TGA was in the range of 26–29%. The amount of the nanocomposites left after TGA measurements (Fig. 1d) was quite comparable to the nominal titania content (75 wt%). UV-visible-DRS absorption spectra was recorded in the range of 220–800 nm to study the optical properties of the as-prepared nanocomposites and SGH-TiO_2_. As shown in Fig. 1e, the 75T/CL nanocomposites and 75T/Norit nanocomposite exhibited absorption in the whole UV-visible region, which is favorable for photocatalysis under visible light irradiation. Whereas, SGH-TiO_2_ exhibited a typical absorption edge in the UV region. The band gap calculated for SGH-TiO_2_ by applying Kubelka–Munk function was ∼3.3 eV (Fig. S6b, ESI†). However, for the nanocomposite the shape of the UV-visible-DRS absorption spectra would not allow to estimate the optical band gap of titania. In order to further evaluate the effect of CL composite on the optical band gap, the nanocomposite was prepared with titania nanoparticles and CL composite in the ratio of 99 : 1. However, the optical band gap of titania remains unchanged (Fig. S6b, ESI†), which indicates that the bulk properties of titania remains same and suggest the surface interaction of titania with the composite. FTIR analysis has been carried out to study the functional groups of 75T/CL and 75T/Norit nanocomposites (Fig. 1f). A broad band emerged in the 75T/CL and 75T/Norit nanocomposites at 580–800 cm^−1^ attributed to Ti–O–Ti bridge stretching modes. However, the IR bands from CL composites were not observed in 75T/CL nanocomposites probably due to high titania content in the nanocomposites. Moreover, the composition of the 75T/CL nanocomposites were determined through elemental analysis and the results are summarized in Table 1 (entry 9–13).

**Fig. 2:**
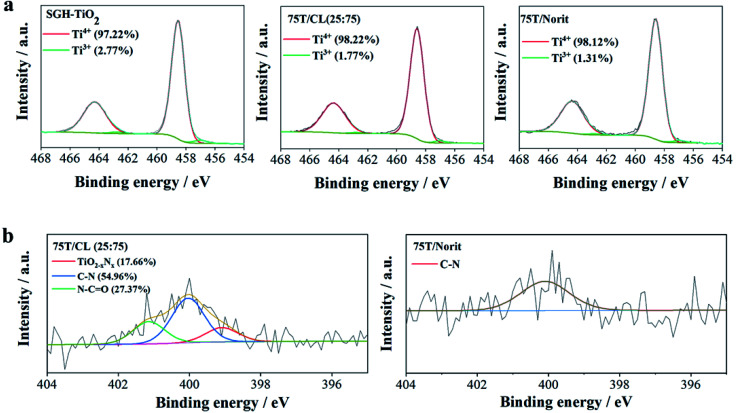
XPS spectra of SGH-TiO_2,_ 75T/CL(25 : 75) and 75T/Norit, (a) Ti 2p (b) N 1s.

X-ray photoelectron spectroscopy (XPS) is a useful technique to study the distinguishing surface functionality of the nanocomposites. The Ti 2p core level spectrum of 75T/CL(25 : 75) nanocomposite can be deconvoluted into four peaks (Fig. 2a), the peaks observed at 458.6 and 464.3 eV assigned to Ti^4+^ 2p_3/2_ and Ti^4+^ 2p_1/2_, respectively. Whereas, the peaks found at 457.0 and 462.8 eV corresponds for Ti^3+^ 2p_3/2_ and Ti^3+^ 2p_1/2_, respectively. Ti^3+^ is generally described as a surface defect of titania, which plays an important role in photocatalysis by preventing electron–hole recombination process, and enhancing the visible light activity.^43^ However, the contribution of Ti^3+^ observed to be very low compared to Ti^4+^ in 75T/CL(25 : 75) (Fig. 2a), the peak area ratio of Ti^3+^/Ti^4+^ was 0.018 : 1. The Ti 2p XPS profile exhibited by SGH-TiO_2_ and 75T/Norit was comparable to 75T/CL(25 : 75). However, in SGH-TiO_2_ the contribution of Ti^3+^ was slightly higher (Fig. 2a), with the peak area ratio of 0.028 : 1 (Ti^3+^/Ti^4+^). Moreover, the presence of nitrogen was clearly evidenced by the N 1s XPS spectra of 75T/CL(25 : 75) and 75T/Norit (Fig. 2b). Three chemical states of nitrogen can be identified after the deconvolution of the N 1s spectrum of 75T/CL(25 : 75) nanocomposite (Fig. 2b). The main signal observed at 400.0 eV can be ascribed to C–N functional group. The second peak appeared at 401.1 eV can be assigned to N–C=O functional group. Whereas, the signal observed at the binding energy of 399.0 eV is probably due to the substitution of oxygen with nitrogen in the framework of the titania, indicating the formation of N–Ti–O bond.^44–46^ This could be beneficial for the visible light photocatalytic activity of 75T/CL(25 : 75) nanocomposite. However, no signals of N element were detected in SGH-TiO_2_. Whereas, N 1s spectrum of 75T/Norit deconvoluted into single peak, at the binding energy of 400.1 eV which corresponds for C–N functional group. The C–N functional group in 75T/Norit may appear due to the atmospheric interaction, as nitrogen was not detected in Norit during XPS and elemental analysis.

**Fig. 3:**
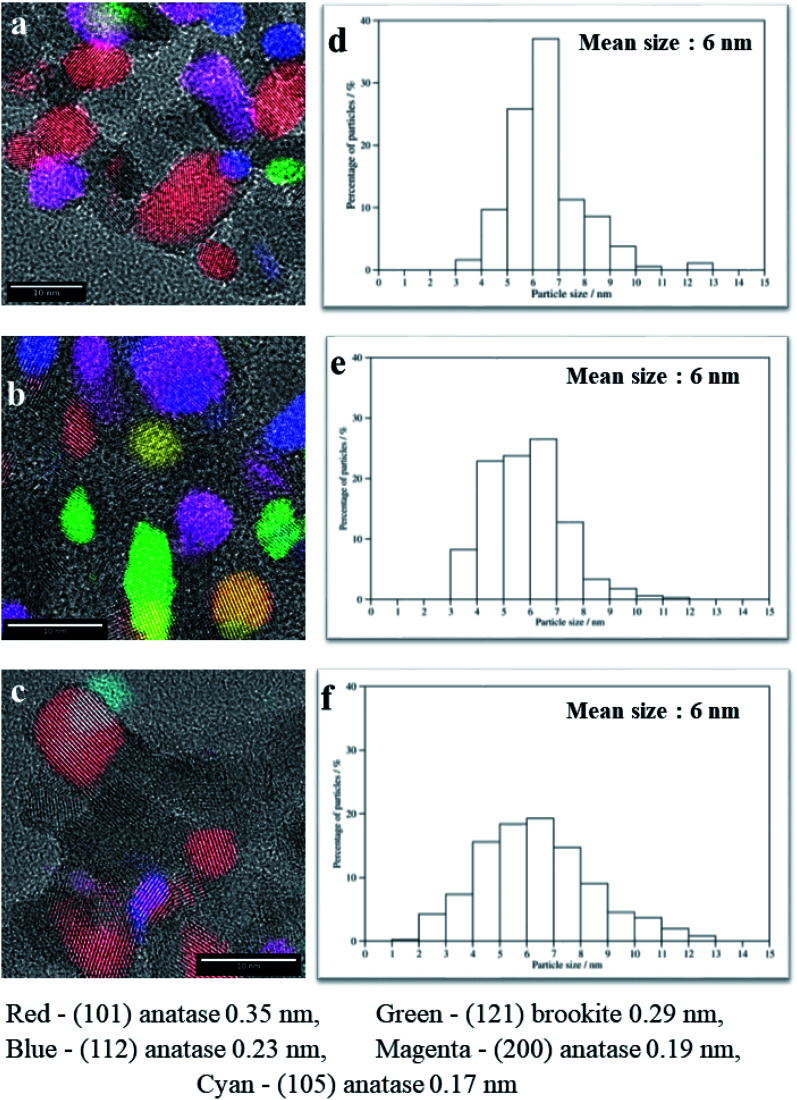
High resolution TEM image of (a) SGH-TiO_2_ (b) 75T/CL(25 : 75) (c) 75T/Norit, particle size distribution in (d) SGH-TiO_2_ (e) 75T/CL(25 : 75) (f) 75T/Norit. (The legend showed the crystal planes and lattice spacing (nm).)

The morphology, average particle size and phase composition of the SGH-TiO_2_, 75T/CL(25 : 75) and 75T/Norit was further corroborated *via* TEM analysis (Fig. 3a–f). The TEM characterization of SGH-TiO_2,_ 75T/CL(25 : 75) and 75T/Norit revealed that SGH-TiO_2_, 75T/CL(25 : 75) and 75T/Norit contain anatase and brookite phase (Fig. 3a–c), Moreover, SGH-TiO_2,_ 75T/CL(25 : 75) and 75T/Norit exhibited the same average particle size *i.e.* 6 nm (Fig. 3d–f). However, the particle size for SGH-TiO_2,_ 75T/CL(25 : 75) and 75T/Norit nanocomposite seems slightly larger than the mean size (Fig. 3a–c), probably due to agglomeration. The results of TEM characterization are in good agreement with the XRD results.

**Fig. 4:**
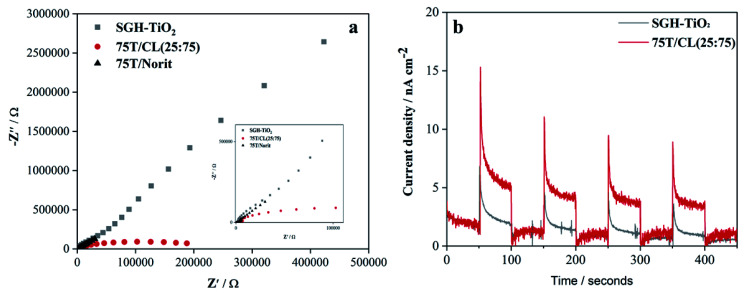
(a) EIS Nyquist plots of SGH-TiO_2,_ 75T/CL(25 : 75) and 75T/Norit (b) transient photocurrent responses SGH-TiO_2_ and 75T/CL(25 : 75).

Electrochemical impedance spectroscopy (EIS) was employed to study the charge transportation ability of photocatalysts, and their Nyquist plots are shown in Fig. 4a. In general, the arc radius of Nyquist plots depicts the resistance of charge transfer between the photocatalysts and electrolyte solution. The arc radius of EIS spectrum of 75T/CL(25 : 75) nanocomposite is smaller than those of SGH-TiO_2_ and 75T/Norit nanocomposite (Fig. 4a), revealing the better charge separation efficiency and a faster interfacial charge transfer^47,48^ on 75T/CL(25 : 75), which could be beneficial for photocatalytic view point. Moreover, the photocurrent response (Fig. 4b) of 75T/CL(25 : 75) is comparatively higher than those of SGH-TiO_2_ under visible light, which further suggest that the 75T/CL(25 : 75) is better able to generate and transfer the photogenerated charge carrier under visible light irradiation. Whereas, 75T/Norit did not show any response when the light was turned on and off (Fig. S7, ESI†), depicting that 75T/Norit was not photoactive under the visible light. Moreover, the noise in the signal observed for 75T/Norit (Fig. S7, ESI†) may correspond to the imperfect contact between the electrode surface and electrolyte.

### 3.3 Photocatalytic activity and selectivity of 75T/CL nanocomposites

#### 3.3.1 Selective oxidation of benzyl alcohol (BnOH) under UV light

**Fig. 5:**
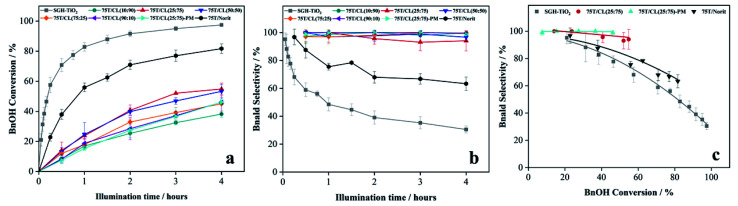
(a) BnOH conversion profile of SGH-TiO_2_, 75T/CL nanocomposites and 75T/Norit nanocomposite as a function of time under UV light (375 nm) (b) Bnald selectivity profile of SGH-TiO_2_, 75T/CL nanocomposites and 75T/Norit nanocomposite as a function time under UV light (375 nm) (c) BnOH conversion *versus* Bnald selectivity plot for SGH-TiO_2_, 75T/CL(25 : 75) nanocomposite and 75T/Norit nanocomposite under UV light (375 nm).

The photocatalytic performance of SGH-TiO_2_, 75T/CL nanocomposites, 75T/Norit and physical mixture (75T/CL(25 : 75)-PM) of SGH-TiO_2_ and CL(25 : 75) was investigated for the selective oxidation of benzyl alcohol (BnOH) to benzaldehyde (Bnald) in acetonitrile under UV light (375 nm). As shown in Fig. 5a, SGH-TiO_2_ and 75T/Norit both exhibited a high activity for BnOH oxidation under UV light, with 97% and 82% BnOH conversion after 4 hours of illumination, respectively. Whereas, different 75T/CL nanocomposites (75T/CL(10 : 90), 75T/CL(25 : 75), 75T/CL(50 : 50), 75T/CL(75 : 25) and 75T/CL(90 : 10)), showed comparatively lower activity for BnOH conversion (38–55%) under UV light (Fig. 5a). Moreover, varying the chitosan and lignin ratio did not cause large difference in the photocatalytic performance of the nanocomposites (Table 3, entries 2–6). Interestingly, the physical mixture of SGH-TiO_2_ and CL(25 : 75) composite (75T/CL(25 : 75)-PM) showed photocatalytic activity comparable to 75T/CL nanocomposites, with 46% BnOH conversion after 4 hours of illumination (Fig. 5a). The high activity of SGH-TiO_2_ may be ascribed to the high oxidizing power of pristine titania under UV light, and the presence of OH groups on the surface of titania (Ti–OH), which may cause the generation of highly reactive ˙OH radicals and results in improved BnOH conversion. In order to evaluate, whether the relatively lower photocatalytic activity of 75T/CL nanocomposites is related to the lower (75 wt%) overall titania content or not, an experiment has been performed with higher (1.35 g L^−1^) photocatalyst (75T/CL(25 : 75) nanocomposite) loading, containing an equivalent titania content as in 1 g L^−1^ of SGH-TiO_2_. BnOH conversion was not significantly increased (Table 3, entry 7) using the same amount of titania. This indicates that the overall titania content might not be the crucial reason of the improved activity of SGH-TiO_2_. Besides that, the formation of blackish-brown suspension in case of 75T/CL nanocomposites may also be related to their lower photocatalytic activity, probably due to the increased opacity and shielding effect, which influence photon absorption. Whereas, the improved activity of 75T/Norit compared to 75T/CL nanocomposites under UV light might be related to its higher specific surface area (239 m^2^ g^−1^). Furthermore, the phase composition and crystallite size of SGH-TiO_2_, 75T/Norit and the 75T/CL nanocomposites were comparable (Table 2, entries 7–13) and probably not the cause for the different photocatalytic activity under UV light. Interestingly, 75T/CL nanocomposites showed high Bnald selectivity (>90%) over the course of reaction compared to SGH-TiO_2_ and 75T/Norit under UV light (Fig. 5b). To ensure that the observed phenomenon of increased selectivity is not caused by the lower activity and thus BnOH conversion *vs.* Bnald selectivity was plotted for SGH-TiO_2_, 75T/Norit, 75T/CL(25 : 75) (a representative sample selected from the 75T/CL nanocomposite series) and 75T/CL(25 : 75)-PM (Fig. 5c). It can be seen that, at a lower BnOH conversion (∼21%) all the photocatalysts exhibited high Bnald selectivity (>90%). However, at a higher BnOH conversion (∼50%) 75T/CL(25 : 75) and 75T/CL(25 : 75)-PM maintained high Bnald selectivity (>90%). The lower Bnald selectivity of SGH-TiO_2_ (68%, see also Table 3, entry 1) might be related to the formation of highly reactive species (h^+^ and ˙OH) by titania under UV light which results in over-oxidation of BnOH. Besides that, the Bnald produced may also compete with BnOH for further oxidation until mineralization. In contrast, the higher Bnald selectivity exhibited by 75T/CL(25 : 75)-PM and 75T/CL(25 : 75) nanocomposite indicate that the presence of CL composite in the reaction medium either as nanocomposite or physical mixture may hinder the mineralization pathway.

**Table 3:** The summary of the photocatalytic oxidation of BnOH over titania and titania/chitosan–lignin (T/CL) nanocomposites

Entries	Photocatalyst	Light	BnOH conv./%	Bnald sel./%	C balance/%
1	SGH-TiO_2_	UV	a58	68	81
2	75T/CL(10 : 90)	UV	38	100	100
3	75T/CL(25 : 75)	UV	55	94	97
4	75T/CL(50 : 50)	UV	53	97	98
5	75T/CL(75 : 25)	UV	45	100	100
6	75T/CL(90 : 10)	UV	46	99	<99
7	b75T/CL(25 : 75)	UV	62	92	95
8	75T/Norit	UV	c56	76	87
9	Phys. mix. (TiO_2_ : CL(25 : 75))	UV	46	99	<99
10	85T/CL(25 : 75)	UV	d59	79	88
11	95T/CL(25 : 75)	UV	e58	75	86
12	99T/CL(25 : 75)	UV	a47	82	92
13	75T/C	UV	52	100	100
14	75T/L	UV	6	100	100
15	SGH-TiO_2_	Visible	0.0	0.0	100
16	75T/CL(10 : 90)	Visible	12	100	100
17	75T/CL(25 : 75)	Visible	19	100	100
18	75T/CL(50 : 50)	Visible	16	100	100
19	75T/CL(75 : 25)	Visible	14	100	100
20	75T/CL(90 : 10)	Visible	15	100	100
21	b75T/CL(25 : 75)	Visible	30	99	<99
22	75T/Norit	Visible	0.0	0.0	100
23	Phys. mix. (TiO_2_ : CL(25 : 75))	Visible	0.0	0.0	100
24	85T/CL(25 : 75)	Visible	17	100	100
25	95T/CL(25 : 75)	Visible	33	100	100
26	99T/CL(25 : 75)	Visible	12	100	100
27	75T/C	Visible	14	100	100
28	75T/L	Visible	0.0	0.0	100
29	75T/CL(25 : 75)	Dark	0.0	0.0	100
30	99T/C	Visible	16	94	99
31	99T/C	UV	f62	67	78
32	No catalyst	UV	0.0	0.0	100
33	No catalyst	Visible	0.0	0.0	100

a Reaction time (15 minutes).

b Catalyst loading (1.35 g L^−1^).

c Reaction time (1 hour).

d Reaction time (2 hours).

e Reaction time (45 minutes).

f Reaction time (30 minutes).

**Fig. 6:**
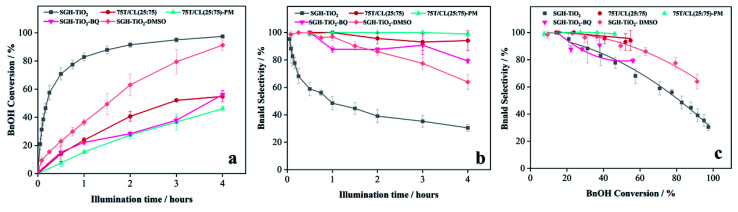
(a) Effect of radical scavenger on BnOH conversion profile of SGH-TiO_2_, as a function of time under UV light (375 nm) (b) effect of radical scavenger on Bnald selectivity profile of SGH-TiO_2_, as a function of time under UV light (375 nm) (c) effect of radical scavenger on BnOH conversion *versus* Bnald selectivity plot for SGH-TiO_2_, under UV light (375 nm).

Besides that, it is hypothesized that CL composite may play a role of radical (˙OH, O_2_^−^˙) scavenger and thereby inhibit the unwanted over-oxidation reactions. To corroborate that hypothesis, the SGH-TiO_2_ catalyzed oxidation of benzyl alcohol was carried out in the presence of radical scavengers (Fig. 6). The addition of dimethylsulfoxide (SGH-TiO_2_–DMSO) as a hydroxyl radical (˙OH) scavenger slowed down the BnOH conversion initially (Fig. 6a). However, after 4 hours of illumination no significant change in the BnOH conversion (91%) was observed. This indicates that ˙OH play a minor role in BnOH oxidation. This is consistent with the fact that it is known from the literature that only limited number of ˙OH could be generated from a UV-irradiated water-saturated titania surface in acetonitrile (as solvent).^49,50^ However, the improved Bnald selectivity in the presence of ˙OH scavenger also indicates the inhibition of a ˙OH-driven unwanted over-oxidation reaction. Whereas, when benzoquinone (BQ) was used as superoxide radical anion (O_2_^−^˙) scavenger the BnOH conversion was almost reduced to half (Fig. 6a), indicating that O_2_^−^˙ is one of the key active species for BnOH oxidation. However, the Bnald selectivity remains essentially unchanged (Fig. 6b). This indicates that O_2_^−^˙ scavenger may hinder the oxidation of BnOH. However, the oxidation of BnOH is still carried out by other oxidizing species. Moreover, these results also suggest that CL composite may play a role of radical scavengers and reduce the BnOH oxidation activity (by scavenging O_2_^−^˙) and improve the Bnald selectivity (by ˙OH scavenging). Besides that, the improvement in Bnald selectivity (>90%) observed for 75T/CL(25 : 75)-PM and 75T/CL(25 : 75) nanocomposite (Fig. 6c), compared to SGH-TiO_2_ (68%) and 75T/Norit (76%) also suggested that there might be an interaction of SGH-TiO_2_ and CL(25 : 75) either as nanocomposite or physical mixture within the reaction medium, which suppresses the mineralization pathway and favours the partial oxidation of BnOH to Bnald.

**Fig. 7:**
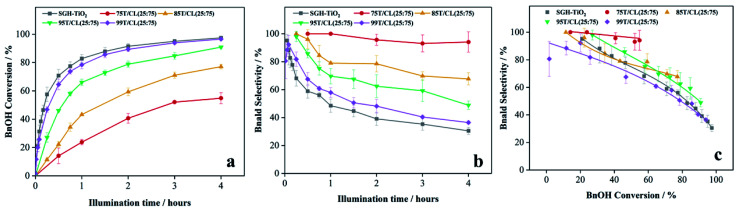
(a) Effect of titania content in nanocomposites on the BnOH conversion profile as a function of time under UV light (375 nm) (b) effect of titania content in nanocomposites on the Bnald selectivity profile as a function time under UV light (375 nm) (c) effect of titania content in nanocomposites on BnOH conversion *versus* Bnald selectivity plot under UV light (375 nm).

Furthermore, the effect of titania content within the nanocomposite on the photocatalytic selective oxidation of BnOH has also been studied. As shown in Fig. 7a, the BnOH conversion increases with an increase in titania content within the nanocomposite. However, the selectivity of Bnald (Fig. 7b and c) was decreased at higher (>75 wt%) titania content (Table 3, entries 10–12). To further investigate the role of chitosan and lignin on the photocatalytic activity of 75T/CL nanocomposites, two new sets of nanocomposites were prepared by coupling titania with chitosan (75T/C) and lignin (75T/L). Interestingly, 75T/C (Table 3 entry 13) showed similar photocatalytic activity in terms of BnOH conversion (52%) and Bnald selectivity (100%) as 75T/CL nanocomposites (Table 3, entries 2–6). However, 75T/L showed negligible BnOH conversion after 4 hours of illumination (Table 3, entry 14). The inactivity of 75T/L nanocomposite might be related to the enhanced UV blocking ability of lignin in the absence of chitosan. Though, this is not a direct proof, it is suggested that lignin may encapsulate the titania in the absence of chitosan and possibly block the UV light and diminished the photocatalytic activity of titania. Recently, lignin has increasingly been used in sunscreen to quench the photocatalytic activity of titania due to its UV shielding properties.^51–53^

#### 3.3.2 Selective oxidation of benzyl alcohol (BnOH) under visible light

**Fig. 8:**
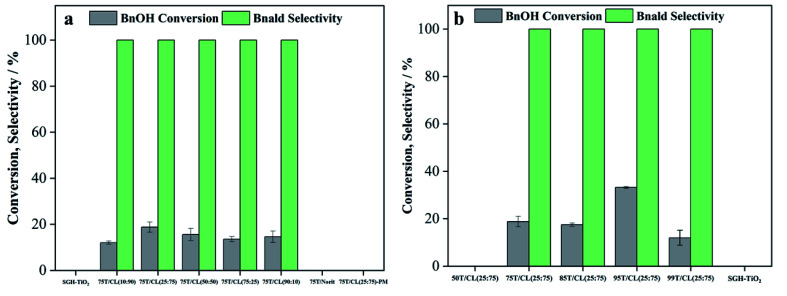
(a) Photocatalytic performance of SGH-TiO_2_, 75T/CL nanocomposites and 75T/Norit nanocomposite for the selective oxidation of BnOH under visible light (515 nm) (b) effect of titania content on the photocatalytic performance of nanocomposites for the selective oxidation of BnOH under visible light (515 nm).

To explore the effect of incident light wavelength, the photocatalytic selective oxidation of benzyl alcohol (BnOH) was also performed under visible light (515 nm). Though, some studies carried out in the past using CdS quantum dots^54^ and Bi_4_O_5_Br_2_ nanoflakes^55^ showed very high selectivity for Bnald (99%) in BnOH oxidation reaction under blue light. However, the current study attempts to utilize far visible region (green light) for the selective oxidation of BnOH without compromising the selectivity of Bnald. Interestingly, 75T/CL nanocomposites were photocatalytically active for the selective oxidation of BnOH (Fig. 8a) under visible light (515 nm). As expected, the BnOH conversion achieved after 4 hours of illumination by 75T/CL nanocomposites under visible light was lower (<20%) compared to UV light. However, all the 75T/CL nanocomposites exhibited high Bnald selectivity (100%) after 4 hours of illumination (Fig. 8a). SGH-TiO_2_, 75T/Norit and 75T/CL(25 : 75)-PM were found to be photocatalytically inactive for the selective oxidation of BnOH under visible light (Fig. 8a). It is suggested that the visible light activity of 75T/CL nanocomposites may be ascribed to the N-doping of titania by the CL composite. The XPS results of the 75T/CL(25 : 75) nanocomposite (a representative sample) showed the substitutional doping of titania (Fig. 2b), which may lead to the visible light activity of 75T/CL. This is consistent with the observation that no such interaction of titania and nitrogen was observed for 75T/Norit and SGH-TiO_2_ which were inactive under visible light. It is likely that the incorporation of nitrogen into the titania framework may results in the formation of a new mid-gap energy state, *i.e.*, the N 2p band above the O 2p valence band, which reduces the band gap of titania.^56^ However, the 75T/CL nanocomposite showed absorption in the whole UV-visible region (Fig. 1e). Therefore, it is difficult to estimate the shift in the optical absorption of titania to the visible light region due to potential N-doping. To further evaluate the potential changes in the band gap of titania by N-doping through CL composites, the UV-visible-DRS absorption spectra has been recorded for nanocomposites with higher titania content (99T/CL(25 : 75), 99T/C). However, only a negligible difference was observed in the band gap of SGH-TiO_2,_ and 99T/CL(25 : 75), 99T/C nanocomposites (Fig. S6b†). It is suggested that N-doping of titania by CL composites leads to surface modification through the bonding of nitrogen,^56^ while the bulk material is still in the intrinsic configuration of pristine titania. Besides that, the doping of nitrogen can possibly suppress the charge recombination efficiency of the photogenerated electron–hole pair, which can contribute to improve the photocatalytic activity and selectivity.^57,58^ The improved charge transfer in 75T/CL(25 : 75) nanocomposite (a representative sample) compared to 75T/Norit and SGH-TiO_2_ is also demonstrated by the EIS Nyquist plots (Fig. 4a) and transient photocurrent response (Fig. 4b) analysis.

Moreover, it was observed that the titania content within the nanocomposites also influences photocatalytic activity under visible light. The BnOH conversion reaches the value of 33% at 95 wt% titania content 95T/CL(25 : 75) after 4 hours of illumination (Fig. 8b). However, further increasing the titania content up to 99 wt% 99T/CL(25 : 75) decreased the BnOH conversion to 12% and did not affect the Bnald selectivity (Table 3, entry 26). The decrease in BnOH conversion at a very high titania content (99 wt%) might be related to the reduced chances of N-doping due to lower CL amount.

### 3.4 Apparent quantum yield (AQY)

The photon flux absorbed by the photocatalytic system was calculated to be 8.70 × 10^−9^ and 11.63 × 10^−9^ Es^−1^ under UV and visible light, respectively. Whereas, the apparent quantum yields (AQY) demonstrated by SGH-TiO_2_, 75T/CL(25 : 75) and 75T/Norit for Bnald production after 4 hours of illumination under UV light were calculated to be 2.3%, 4.1% and 4.1%, respectively. On the other hand, the AQY observed for 75T/CL(25 : 75) under visible light was 1.1%. The obtained AQY are comparable to the reported efficiency of Pd-deposited CdS–TiO_2_ composite (1.4%)^59^ and Au/TiO_2_ nanorods (3.4%)^60^ for the selective oxidation of benzyl alcohol at 540 nm and >420 nm, respectively.

### 3.5 Stability and reusability of 75T/CL(25 : 75) nanocomposite

**Fig. 9:**
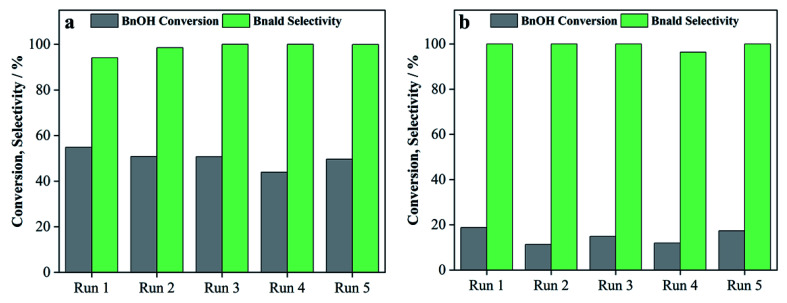
The recycling of 75T/CL(25 : 75) nanocomposite for the selective oxidation of BnOH under (a) UV light (375 nm) irradiation (b) visible light (515 nm) irradiation.

In order to test the stability and reusability of the photocatalyst, leaching of titanium from the nanocomposite during photocatalytic reaction was studied by XRF analysis. However, no titanium leaching was observed for 75T/CL(25 : 75) according to XRF analysis under UV and visible light (Fig. S8†). Moreover, UV-visible absorption spectra were recorded to assess the stability of the 75T/CL(25 : 75) nanocomposite under dark conditions and light (UV and visible) irradiation in acetonitrile indicating the leaching of components of 75T/CL(25 : 75) nanocomposite (Fig. S9†). According to GC-MS analysis, traces of acetamide, 9-octadecenamide, oleanitrile, hexadecanamide, tetradecanamide and 2,4-dimethyl-benzaldehyde were detected in the liquid phase after leaching experiments under dark and light (UV and visible) irradiation. The 2,4-dimethyl-benzaldehyde may be observed from the fragmentation of lignin. Whereas, the rest of the compounds may be formed from the depolymerization of chitosan or originally present in the chitosan as oligomers impurity. To investigate the reusability of the 75T/CL(25 : 75) nanocomposite, recycling experiments for the selective oxidation of BnOH were carried out under the UV and visible light, and the results are shown in Fig. 9a and b, respectively. 75T/CL(25 : 75) nanocomposite selectively oxidize BnOH to Bnald without substantial drop in activity even after five runs under the UV and visible light.

## 4. Conclusions

Chitosan–lignin (CL) composites with multiple functional groups were prepared through a hydrothermal method. Moreover, a series of nanocomposites (T/CL) was synthesized by combining titania with CL using sol–gel and hydrothermal based methods. This coupling of titania with CL not only enhanced the selectivity to benzaldehyde (Bnald) in benzyl alcohol (BnOH) oxidation reaction under UV light but also triggers the visible light activity. The synergy between chitosan and titania plays a crucial role for the visible light activity. It is suggested that the visible light activity of T/CL nanocomposites could be related to the introduction of nitrogen from chitosan into titania. Whereas, the improved performance of T/CL nanocomposites under UV light possibly be related to the radical scavenging properties of CL composites. It can thus be concluded that nanocomposites from titania and sustainable biomass-based materials improved the photocatalytic performance of titania due to several non-intuitive advantages such as an indirect improvement in light harvesting (*via* N-doping) and increased selectivity through mediation (scavenging) of reactive unselective radical species.

## Conflicts of interest

The authors declare no conflict of interest.

## Supplementary Material

RA-011-D1RA06500A-s001
