# Correction: Titania/chitosan–lignin nanocomposite as an efficient photocatalyst for the selective oxidation of benzyl alcohol under UV and visible light

**DOI:** 10.1039/d2ra90004a

**Published:** 2022-02-09

**Authors:** Ayesha Khan, Michael Goepel, Wojciech Lisowski, Dariusz Łomot, Dmytro Lisovytskiy, Marta Mazurkiewicz-Pawlicka, Roger Gläser, Magdalena Warczak, Juan Carlos Colmenares

**Affiliations:** Institute of Physical Chemistry, Polish Academy of Sciences Warsaw 01-224 Poland akhan@ichf.edu.pl jcarloscolmenares@ichf.edu.pl; Institute of Chemical Technology, Leipzig University Leipzig 04103 Germany roger.glaeser@uni-leipzig.de; Faculty of Chemical and Process Engineering, Warsaw University of Technology Warsaw 00-645 Poland

## Abstract

Correction for ‘Titania/chitosan–lignin nanocomposite as an efficient photocatalyst for the selective oxidation of benzyl alcohol under UV and visible light’ by Ayesha Khan *et al.*, *RSC Adv.*, 2021, **11**, 34996–35010. DOI: 10.1039/D1RA06500A

The authors regret the omission of one of the authors, Magdalena Warczak, from the original manuscript. The corrected author list is as shown above.

The Royal Society of Chemistry apologises for these errors and any consequent inconvenience to authors and readers.

## Supplementary Material

