# Supplementary material for: Titania/chitosan–lignin nanocomposite as an efficient photocatalyst for the selective oxidation of benzyl alcohol under UV and visible light
Source: RSC Adv. 2021 Oct 28;11(55):34996–5010. doi: 10.1039/d1ra06500a (PMC9042820; doi:10.1039/d1ra06500a)
Supplement: RA-011-D1RA06500A-s001 [file RA-011-D1RA06500A-s001.pdf]

Electronic Supplementary Material (ESI) for RSC Advances.
This journal is © The Royal Society of Chemistry 2021

Supplementary Information

Titania/Chitosan-Lignin nanocomposite as an efficient photocatalyst for the selective oxidation of benzyl alcohol under UV and visible light

Ayesha Khan,*[a] Michael Goepel,[b] Wojciech Lisowski,[a] Dariusz Łomot,[a] Dmytro Lisovytskiy,[a] Marta Mazurkiewicz-Pawlicka,[c] Roger Gläser,*[b] Juan Carlos Colmenares*[a]

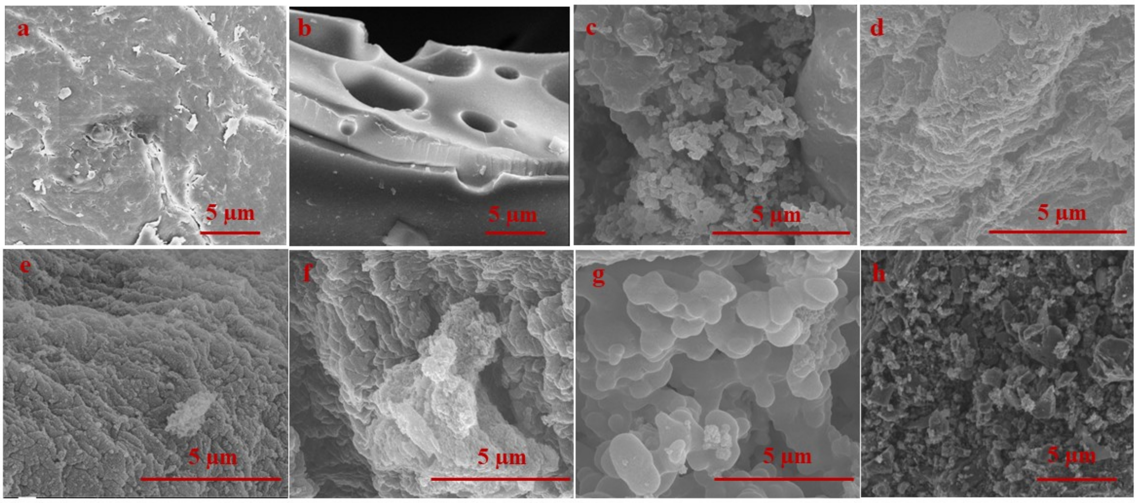

Fig. S1 Scanning electron microscope (SEM) images of a) Chitosan, b) Lignin, c) CL(10:90), d) CL(25:75), e) CL(50:50), f) CL(75:25), g) CL (90:10), h) Norit

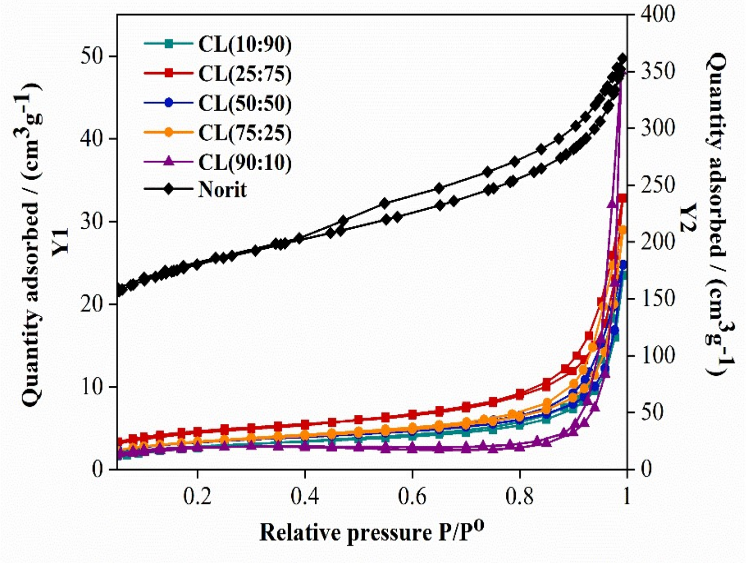

Fig. S2 Nitrogen adsorption-desorption isotherms of CL composites and Norit (Quantity adsorbed by CL composites displayed on Y1 axis and quantity adsorbed by Norit displayed on Y2 axis).

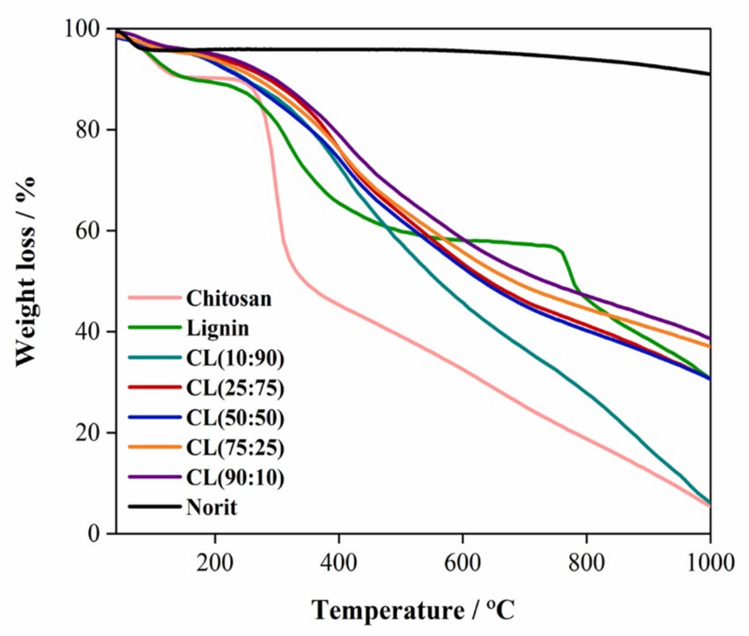

Fig. S3 Thermogravimetric analysis (TGA) curves of CL composites and Norit under N_2 atmosphere.

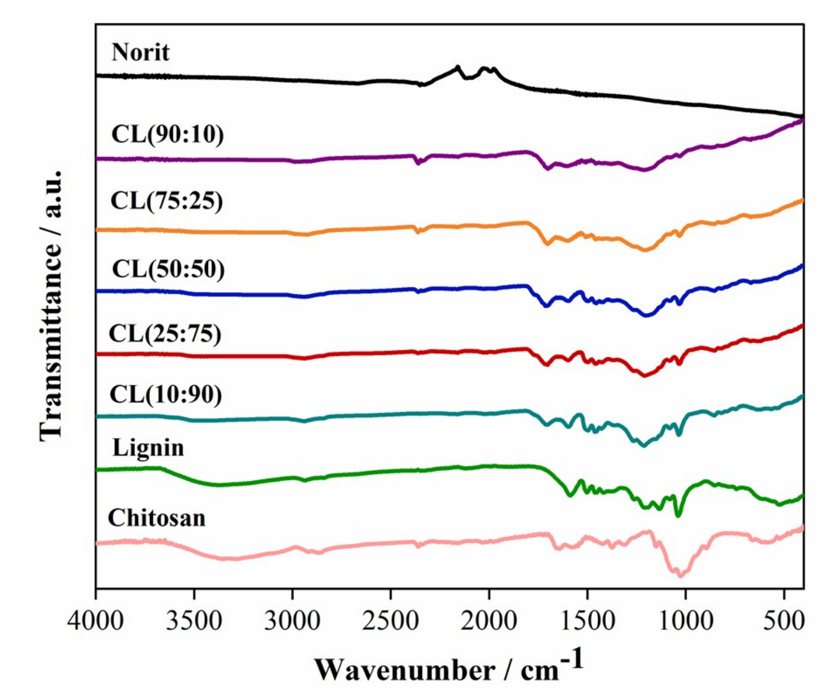

Fig. S4 FTIR spectra of chitosan, lignin, Norit and CL composites.

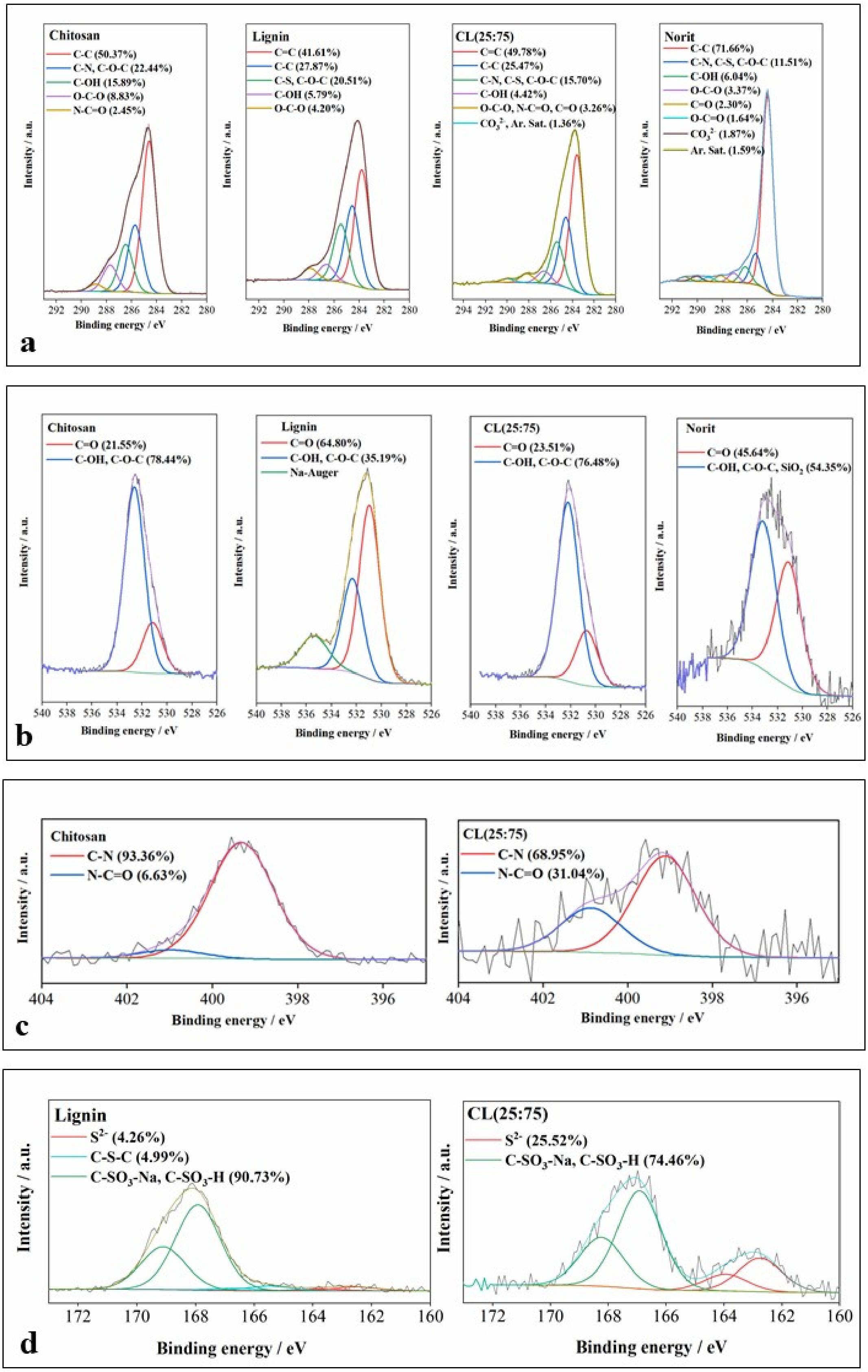

Fig. S5 XPS spectra of chitosan, lignin, CL(25:75) and Norit a) C 1s, b) O 1s c) N 1s d) S2p

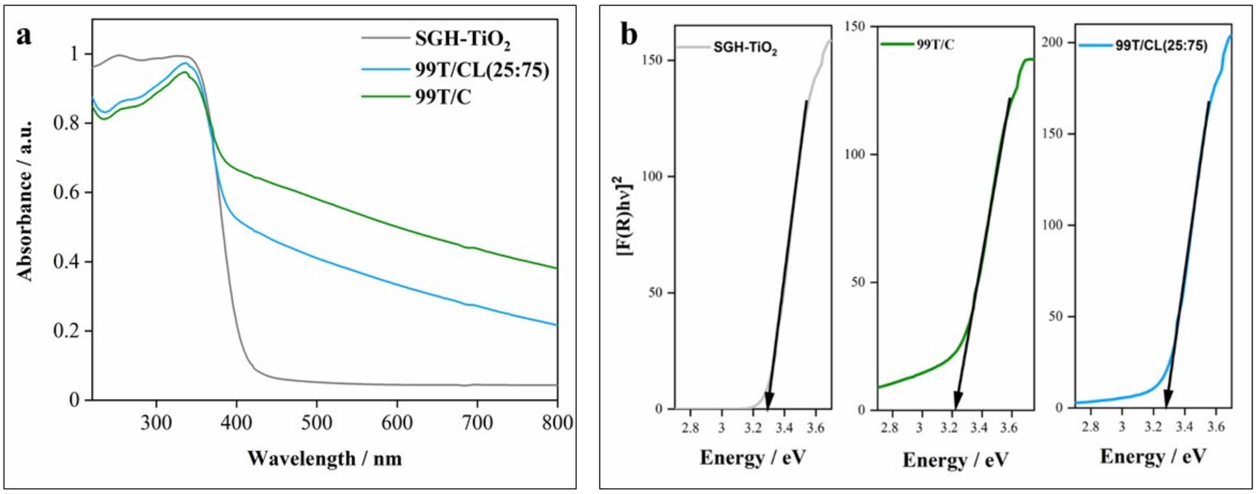

Fig. S6 a) UV-Visible DRS absorption spectra of SGH-TiO_2, 99T/CL (25:75) and 99T/C. b) Tauc plot for SGH-TiO_2, 99T/C, 99T/CL(25:75).

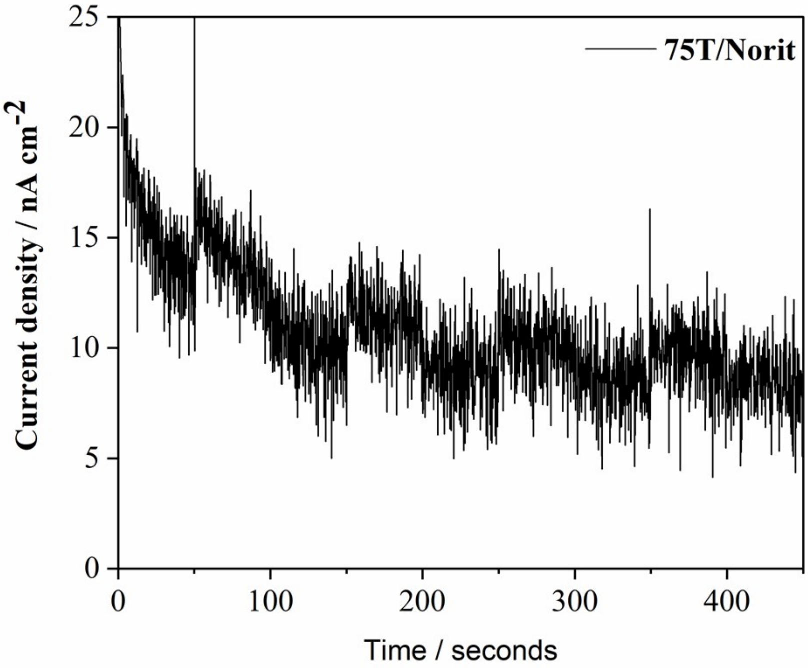

Fig. S7 Transient photocurrent response of 75T/Norit

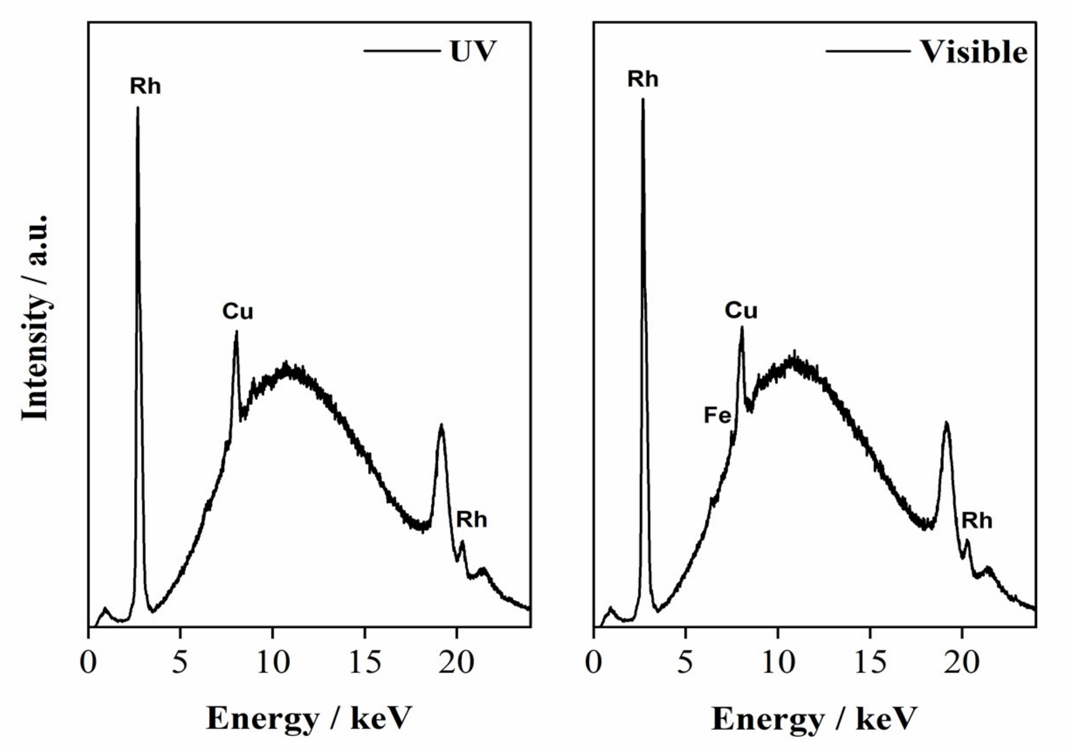

Fig. S8 XRF analysis of BnOH solution catalyzed over 75T/CL(25:75) nanocomposite.

There are no visible traces of Ti on XRF spectra of the samples. The peaks observed corresponds to Rh from Rh lamp, Fe and Cu peaks which is a finger print of the spectrometer.

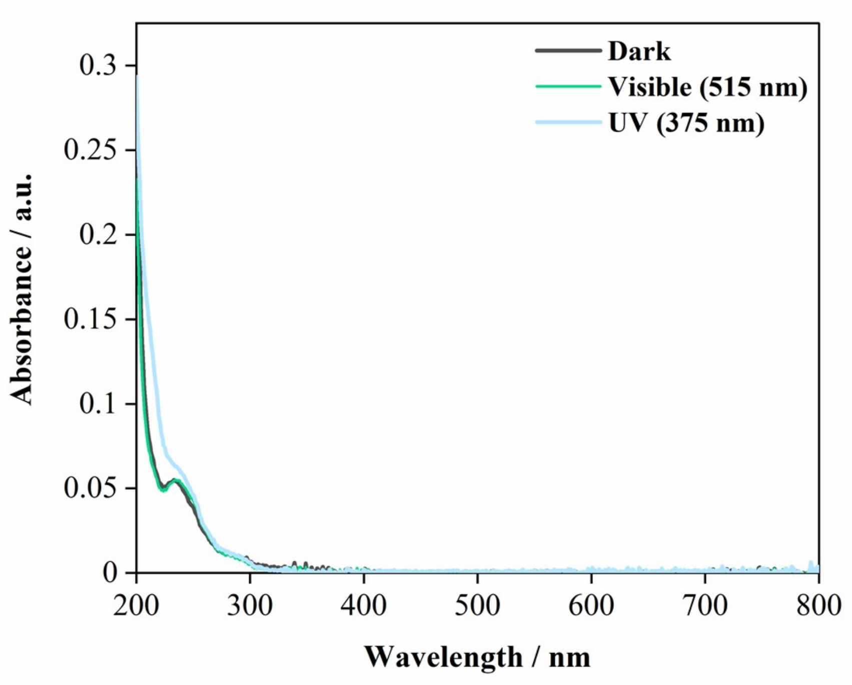

Fig. S9 UV-Visible absorption spectra of 75T/CL(25:75) extracts in acetonitrile.

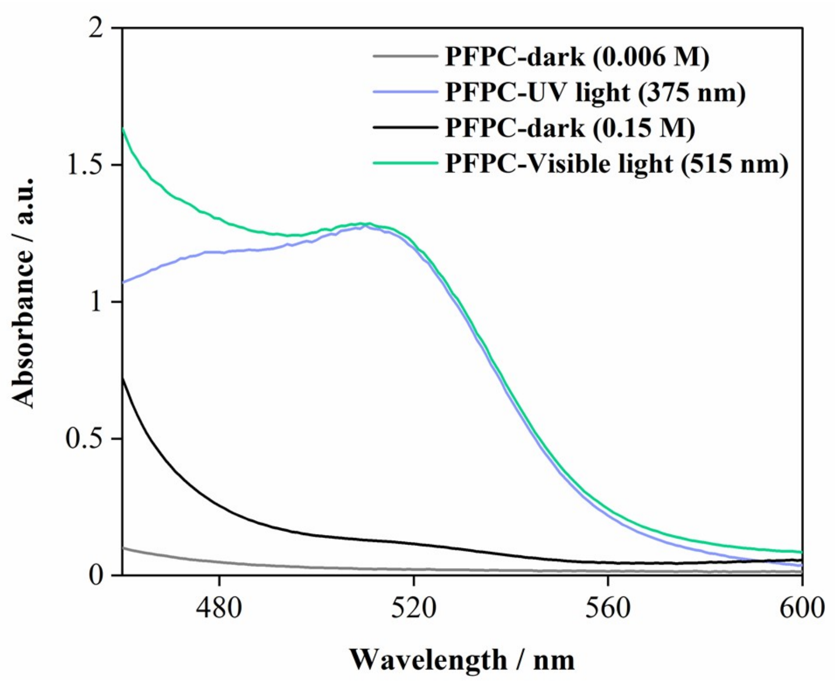

Fig. S10 UV-Visible absorption spectrum of potassium–ferrioxalate phenanthroline complex (PFPC).
